# Specific Oscillatory Power Changes and Their Efficacy for Determining Laterality in Mesial Temporal Lobe Epilepsy: A Magnetoencephalographic Study

**DOI:** 10.3389/fneur.2021.617291

**Published:** 2021-02-09

**Authors:** Yuta Tanoue, Takehiro Uda, Hideyuki Hoshi, Yoshihito Shigihara, Toshiyuki Kawashima, Kosuke Nakajo, Naohiro Tsuyuguchi, Takeo Goto

**Affiliations:** ^1^Department of Neurosurgery, Graduate School of Medicine, Osaka City University, Osaka, Japan; ^2^Precision Medicine Centre, Hokuto Hospital, Obihiro City, Japan; ^3^Precision Medicine Centre, Kumagaya General Hospital, Kumagaya, Japan; ^4^Department of Neurosurgery, Faculty of Medicine, Kindai University, Osaka, Japan

**Keywords:** magnetoencephalography, oscillatory power change, mesial temporal lobe epilepsy, lateralizing sign, machine learning

## Abstract

Appropriate determination of the epileptic focus and its laterality are important for the diagnosis of mesial temporal lobe epilepsy (MTLE). The aims of this study are to establish a specific oscillatory distribution and laterality of the oscillatory power in unilateral MTLE with frequency analysis of magnetoencephalography (MEG), and to confirm their potential to carry significant information for determining lateralization of the epileptic focus. Thirty-five patients with MTLE [left (LtMTLE), 16; right (RtMTLE), 19] and 102 healthy control volunteers (CTR) were enrolled. Cortical oscillatory powers were compared among the groups by contrasting the source images using a one-way ANOVA model for each frequency band. Further, to compare the lateralization of regional oscillatory powers between LtMTLEs and RtMTLEs, the laterality index (LI) was calculated for four brain regions (frontal, temporal, parietal, and occipital) in each frequency band, which were compared between patient groups (LtMTLE, RtMTLE, and CTR), and used for machine learning prediction of the groups with support vector machine (SVM) with linear kernel function. Significant oscillatory power differences between MTLE and CTR were found in certain areas. In the theta to high-frequency oscillation bands, there were marked increases in the parietal lobe, especially on the left side, in LtMTLE. In the theta, alpha, and high-gamma bands, there were marked increases in the parietal lobe, especially on the right side in RtMTLE. Compared with CTR, LIs were significantly higher in 24/28 regions in LtMTLE, but lower in 4/28 regions and higher in 10/28 regions in RtMTLE. LI at the temporal lobe in the theta band was significantly higher in LtMTLE and significantly lower in RtMTLE. Comparing LtMTLE and RtMTLE, there were significant LI differences in most regions and frequencies (21/28 regions). In all frequency bands, there were significant differences between LtMTLE and RtMTLE in the temporal and parietal lobes. The leave-one-out cross-validation of the linear-SVM showed the classification accuracy to be over 91%, where the model had high specificity over 96% and mild sensitivity ~68–75%. Using MEG frequency analysis, the characteristics of the oscillatory power distribution in the MTLE were demonstrated. Compared with CTR, LIs shifted to the side of the epileptic focus in the temporal lobe in the theta band. The machine learning approach also confirmed that LIs have significant predictive ability in the lateralization of the epileptic focus. These results provide useful additional information for determining the laterality of the focus.

## Introduction

Epilepsy is one of the most common neurological disorders, and surgery is one of the treatment options. Mesial temporal lobe epilepsy (MTLE) is known to be particularly responsive to surgery (1). In a previous randomized trial, 58% of the patients with MTLE achieved seizure-free status from impaired awareness seizures in the surgical group, compared to 8% in the medical group, at 1 year (2). In another report, long-term seizure-free rates of MTLE were 47% at 5 years and 38% at 10 years, in the surgically treated group (3). On the other hand, the cause of re-operation due to a poor seizure outcome after surgery has been reported to be incorrect localization or insufficient resection of the epileptogenic zone (4). Especially in the case of MTLE, appropriate determination of the laterality of the epileptic focus is important for successful surgical treatment (5). Laterality is determined based on multimodal assessments pertaining to specific seizure semiology, the dominant side of interictal epileptic discharges on electroencephalography (EEG), unilateral hippocampal sclerosis or atrophy on magnetic resonance imaging (MRI), and unilateral hypo-accumulation on fluorodeoxyglucose positron emission tomography (FDG-PET) or single-photon emission computed tomography (SPECT). Each of these assessments provides information regarding the laterality of the epileptic focus, which is called a “lateralizing sign” or “lateralizing value” (5–8). Although accumulated “lateralizing signs” from multimodal information such as structural and physiological testing and even ictal video EEG findings are available, it is sometimes challenging to determine the laterality of the epileptic focus.

Magnetoencephalography (MEG) is another modality that provides information about lateralizing signs and possesses several advantages. First, MEG can directly and non-invasively measure the electrophysiological activity of the entire brain. Second, MEG has an excellent temporal resolution of <1 ms. Third, signals obtained by MEG are not affected by the conductivity of tissues over the brain, such as the scalp, skull, and cerebrospinal fluid. This leads to the characteristics of MEG providing spatially less distorted information than EEG, when evaluating signals from the same source (9). In general, MEG is sensitive to primary currents running parallel to the brain surface, while it has limited sensitivity to those running radially. Because EEG has contrasting characteristics, the two modalities are used complementarily (10, 11). Conventionally, MEG data have been used to estimate the source of epileptic discharges using equivalent current dipole (ECD) analysis. In ECD analysis, the epileptic discharge is assumed to be caused by electric current in the small area around the epileptic focus, and the location of the ECD is regarded as the lateralizing sign. Although the utility and reliability of the ECD method to determine the laterality of the epileptic focus in patients with MTLE are well-established, the method is often arbitrary, time-consuming, and skill-dependent on the analyst (12–14). The time of analysis was manually selected by observing the MEG spikes, the obtained isocontour map, and the findings from simultaneous EEG recordings. In addition, the sensor selection used for the analysis depends on the analyst. In our institute, it usually takes 30–60 min for recoding and requires at least a few hours for analysis of each participant. It was also found that ECD methods do not always work well with patients who show few epileptic spikes, because the analysis depends on the visual inspection of the epileptic discharges.

Frequency analysis is another method for analyzing the MEG data. It provides resting-state neural oscillatory power at each location in the whole brain. Because of its lower arbitrariness, it is suitable for objective and automated analyses. Recently, specific changes of the oscillatory power in various diseases have been reported for MEG (15–18), as well as functional MRI (19). Sakamoto et al. reported oscillatory changes in the ischemic brain on MEG using standardized low-resolution brain electromagnetic tomography (sLORETA) (15). They reported that the MEG of patients with unilateral internal carotid artery occlusive disease showed statistically significant laterality in the affected middle cerebral artery regions. If a specific oscillatory distribution or laterality of the oscillatory power in unilateral MTLE can be established, it would be helpful for the diagnosis of MTLE from an additional perspective with other conventional modalities.

The aims of the present study using frequency analysis of MEG were to demonstrate that: (i) regional oscillatory power across whole brain was different between healthy control volunteers (CTR) and patients with left or right MTLE, and that (ii) the difference in the reginal oscillatory power has additive value to conventional analysis, and could be used as one of the “lateralizing signs” to determine the side of epileptic focus in patients with MTLE. The methods proposed here would provide additional “lateralizing signs” to improve the plausibility of the determination of laterality of the epileptic focus.

## Materials and Methods

### Patients and Database

This study was conducted in accordance with the Declaration of Helsinki, and was approved by the Ethics Committee of Hokuto Hospital (approval No. 1008) and the Ethics Committee of Osaka City University Hospital (approval No. 4103). The MEG data of patients with MTLE at Osaka City University Hospital recorded between 2006 and 2019 were retrospectively reviewed. Only patients with typical unilateral MTLE were selected from the MEG database at Osaka City University to learn their typical oscillatory power distribution. To identify typical patients with unilateral MTLE, their seizure semiology, interictal/ictal EEG findings, MRI, FDG-PET, and SPECT were considered. Patients with a history of encephalitis, genetic disorder, suspected multifocal epileptogenicity, and bilateral MTLE were excluded.

Sixteen patients with left MTLE (LtMTLE) (9 females; age range, 14–56 years; mean ± SD age, 37 ± 10.9 years) and 19 patients with right MTLE (RtMTLE) (12 females; age range, 8–71 years; mean ± SD age, 34 ± 14.3 years) were enrolled in this study. Detailed clinical information of the patients is presented in Table 1.

**Table 1:** Patients' characteristics.

**Case no**.	**Sex**	**Age (y) at MEG**	**Age (y) at onset**	**Epilepsy duration (y)**	**Side**	**EEG**	**MRI**	**FDG-PET**	**ECD**	**Linear-SVM**	**ICE**	**Surgery**	**Histology**	**Engel**
1	M	71	35	36	R	R	HS	R	No spike	CTR	N	SAH	HS	1a
2	M	31	23	8	R	R	HS	R	R	R	N	SAH	HS	1d
3	F	33	7	26	R	R	HS	R	R	R	N	SAH	HS	1a
4	F	39	30	30	R	R	HS	R	R	R	N	SAH	HS	1a
5	F	38	20	18	R	R	HS	R	No spike	CTR	N	SAH	HS	1a
6	M	36	20	16	R	bilateral	HS	R	Bilateral	R	N	SAH	HS	2c
7	F	20	10	10	R	R	HS	R	R	R	N	SAH	HS	1b
8	F	36	24	12	R	R	Negative	R	R	R	N	SAH	HS	1a
9	M	40	12	28	R	R	Negative	Negative	No spike	R	Y	SAH	HS	1a
10	M	31	21	10	R	R	HS	R	no spike	CTR	N	SAH	HS	1a
11	F	17	15	2	R	R	HS	R	No spike	R	N	SAH	HS	1a
12	M	22	15	6	R	R	HS	R	R	CTR	N	SAH	noHS	1a
13	M	22	18	4	R	R	AE	R	R	R	Y	ATL	noHS	1d
14	F	44	30	14	R	R	Negative	R	No spike	CTR	N	ATL	noHS	1b
15	F	44	19	25	R	R	Negative	Negative	R	R	Y	ATL	noHS	3a
16	F	8	3	5	R	R	HS	R	R	R	N	ATL	FCD	1a
17	F	54	49	5	R	R	AE	Negative	R	R	N	SAH	noHS	1b
18	F	22	16	6	R	R	Negative	R	R	R	–	–	–	
19	F	30	4	15	R	R	Negative	R	R	CTR	–	–	–	
20	F	42	25	17	L	L	HS	L	L	L	N	SAH	HS	1a
21	F	36	20	16	L	L	HS	L	Bilateral	L	N	SAH	HS	1a
22	F	35	17	18	L	L	HS	L	L	L	N	SAH	HS	1a
23	M	30	2	28	L	L	HS	L	L	L	N	SAH	HS	1a
24	M	25	20	5	L	Bilateral	HS	L	L	L	N	SAH	HS	1a
25	M	38	19	19	L	L	HS	L	L	L	N	SAH	HS	1a
26	F	52	12	40	L	Bilateral	HS	L	Bilateral	CTR	Y	SAH	HS	1a
27	M	56	6	50	L	L	HS	L	L	CTR	N	SAH	HS	1a
28	F	14	8	6	L	L	FCD	L	L	L	N	SAH	FCD	1a
29	F	22	20	2	L	L	Negative	L	L	L	N	SAH	HS	1a
30	M	45	14	31	L	L	DNT	L	L	L	N	SAH	noHS	1a
31	M	38	2	36	L	L	HS	L	No spike	L	–	–	–	
32	F	49	6	43	L	L	Negative	L	No spike	CTR	Y	SAH	HS	1b
33	F	41	8	33	L	L	HS	L	L	L	N	SAH	HS	2b
34	M	37	24	13	L	L	HS	L	Bilateral	L	N	SAH	HS	1b
35	F	36	3	33	L	L	HS	L	L	CTR	N	SAH	HS	2c

*MEG, magnetoencephalography; EEG, electroencephalography; MRI, magnetic resonance imaging; FDG-PET, fluorodeoxyglucose positron emission tomography; ECD, equivalent current dipoles; SVM, support vector machine; CTR, healthy control volunteers; ICE, intracranial electrode; Engel, Engel epilepsy surgery outcome scale; M, male; F, female; R, right; L, left; Y, performed; N, not performed; HS, hippocampal sclerosis; noHS, no hippocampal sclerosis; AE, amygdala enlargement; FCD, focal cortical dysplasia; DNT, dysembryoplastic neuroepithelial tumor; SAH, selective amygdalohippocampectomy; ATL, anterior temporal lobectomy*.

As a baseline dataset (CTR), the Hokuto102 database (https://www.hokuto7.or.jp/hospital/lang/english-home/meaw/) provided by Hokuto Hospital was used. The database consisted of 5-min resting-state MEG recordings with eyes closed, acquired from 102 healthy volunteers (54 females; age range, 22–75 years; mean ± SD age, 44 ± 14.2 years).

### MEG Protocol

Spontaneous neural oscillations (i.e., resting-state brain activity) were recorded for 5 min using a 160-channel whole-head type magnetoencephalography system (MEG vision PQ1160C; Yokogawa, Kanazawa, Japan). During the scan, patients were asked to remain calm in the supine position with their eyes closed in a magnetically shielded room. The scanning conditions were controlled to be as consistent and comfortable as possible. The MEG system had a magnetic field resolution of 4 fT/√Hz in the white-noise region. The sensor and reference coils were gradiometers with a diameter of 15.5 mm and a baseline of 50 mm, and each pair of sensor coils was separated by a distance of 23 mm. MEG data were recorded through a 0.3–200-Hz bandpass filter with a sampling rate of 1,000 Hz. To co-register MEG source images with individual structural MRI, three fiducial magnetic marker coils were placed on the patient's face (5 mm above the nasion, and bilaterally 10 mm in front of the tragus) during the MEG scan. Individual structural MRI images were acquired using a 3.0-T scanner (Phillips Achieva 3.0T, Philips Healthcare, Amsterdam, The Netherlands) with a standard head coil, with three fiducial markers (Medtronic Surgical Navigation Technologies Inc., Broomfield, CO, USA) placed at the same position as the magnetic marker coils.

The CTR dataset was acquired under identical conditions as for MTLEs, but at a different MEG site (Hokuto Hospital, Obihiro, Japan). The MEG machine, experimental setups, and recording protocols were identical for the MTLEs and CTRs.

### MEG Analysis

#### Preprocessing

MEG data were analyzed offline using the software package SPM-12 (Wellcome Trust Center for Neuroimaging, London, UK; https://www.fil.ion.ucl.ac.uk/spm/) and the MEAW system (https://www.hokuto7.or.jp/hospital/lang/english-home/meaw/). For ease of analysis, the continuous MEG data were divided into 10-s segments. Epochs in which the magnetic signal exceeded 6,000 fT were discarded, and all of the remaining epochs were used for further analysis. Since the experimental environment generated a utility frequency, an optimized band-stop filter was applied to the epoched data (the utility frequency differed between the sites as: 60 Hz for MTLEs and 50 Hz for CTRs). The filtered data were used directly for source-level analyses. The MEG data were evaluated at the source level, because sensor-level data were biased by the heterogeneity of the distance between the sensor and the brain surface. To identify the locations of the brain producing the resting-state background activity, the source inversion procedures were applied to the oscillation components of delta (0–3 Hz), theta (4–7 Hz), alpha (8–12 Hz), beta (13–25 Hz), gamma (low gamma, 26–40 Hz; high gamma, 41–80 Hz), and high-frequency oscillation (HFO; 81–120 Hz), separately. First, a cortical mesh with 8,196 vertices was created using the “normal” mode of the mesh generation function in SPM-12. The co-registration of MRI images and MEG sensor locations was performed using an iterative closest point algorithm (20). Forward modeling was performed for the whole brain using a single-shell model with normalized individual anatomical MRI images. Source inversion was performed using a maximal smoothness algorithm with a spatially coherent source model [i.e., COH algorithms implemented in SPM-12 (21)], which is similar to sLORETA (22). The COH algorithm is a commonly used source inversion algorithm and is often used in clinical environments (11, 23). Inversion was performed for the bandpass-filtered signal for each frequency band (from delta to HFO), without any source priors. A series of Morlet wavelet projectors (i.e., oscillatory powers) was generated, summarizing the inverted intensity (i.e., energy) in each trial and each band of interest (from delta to HFO). The results were then averaged over trials, which enabled the localization of background activity. The averaged power was projected onto the source space to generate the resulting source images. The source images were then taken for a second (group)-level analysis.

#### Factorial Design Analysis (Evaluation 1—MTLE Patients vs. Normal Database)

For the second-level analysis, the cortical oscillatory powers were compared between the groups (LtMTLE, RtMTLE, and CTR) by contrasting the source images using a one-way ANOVA model (factorial modeling function implemented in SPM-12) for each frequency band. To take into account the influence of participants' ages on the results, age was included as a nuisance covariate of the ANOVA model. The images were contrasted for LtMTLEs vs. CTRs, RtMTLEs vs. CTRs, and LtMTLEs vs. RtMTLEs. The source locations of peak level activations at a significance threshold of *p* [corrected for family wise error (*FEW*)] = 0.05. The cortical areas at which the peaks of the estimated sources were located were identified on the Montreal Neurological Institute (MNI) template using MRIcron. (http://www.mccauslandcenter.sc.edu/mricro/mricron).

#### Laterality Analysis (Evaluation 2—Laterality Index)

Next, to compare the lateralization of regional oscillatory powers between LtMTLEs and RtMTLEs, the laterality of power was calculated using the laterality index (LI) in four brain regions (frontal, temporal, parietal, and occipital) in each frequency band (from delta to HFO). The brain regions were defined by WFU_PickAtlas (24, 25), which generates ROI masks based on the Talairach Daemon database (26, 27). The source images were averaged within four regions using the spm_summarize function (implemented in SPM-12) with the ROI masks. LI was calculated using the well-known basic formula (28, 29):

(1) $$\begin{array}{r} LI=\frac{P_{L}^{fr}-\;P_{R}^{fr}}{P_{L}^{fr}+\;P_{R}^{fr}} \end{array}$$

where P represents the regional oscillatory power (output from the spm_summarize function) in the appropriate hemisphere (L or R) of a given frequency band (*f*), and in a given region (*r*). The LI ranges from −1 to 1, indicating right-to-left dominance. It has been reported that CTR shows significant lateralization (i.e., LIs are not supposed to be zero for the CTR group) (28). Since the aim was to assess the disease-related changes in LIs between LtMTLEs and RtMTLEs, the averaged regional LIs for the CTR group were subtracted from the LIs for the MTLE groups (i.e., LIs of the MTLE group were baseline-corrected). Baseline-corrected LIs were compared using two-way ANOVA with a 2 (Group: LtMTLE and RtMTLE) × 4 (region: frontal, temporal, parietal, and occipital) full factorial design. The results of the ANOVA were evaluated using a non-parametric bootstrapping approach. First, a two-way ANOVA was performed, and *F statistics* (*F*_original_) were calculated using the original dataset. Next, all samples were resampled with replacement across all conditions and levels (i.e., regardless of the group and/or region) 20,000 times, and *F* statistics (*F*_resampled_) were calculated for each resampled dataset. The percentage of *F*_resampled_ being larger or smaller than *F*_original_ (the smaller value) was taken as the significance level for the *F* statistics. Additionally, for each frequency band and each region, baseline-corrected LIs were tested using the one-sample *t*-test against zero (i.e., compared to CTRs), and compared between LtMTLEs and RtMTLEs using the two-sample *t*-test. Bootstrapping approaches were used to evaluate the differences between LtMTLEs and RtMTLEs. For each group, LIs were resampled with replacement 20,000 times across the patients (16 patients in LtMTLEs and 19 patients in RtMTLEs), and the percentage of the resampled differences, being larger or smaller than 0 (the smaller value), was taken as the significance level for the one-sample *t*-test against zero. The differences in the averaged LIs between LtMTLEs and RtMTLEs were calculated for each iteration, and the percentage of the group differences, being larger or smaller than 0 (the smaller value), was taken as the significance level for the two-sample *t*-test between the groups. The false detection rate (FDR) was controlled using the Benjamini and Hochberg method (30).

#### Machine Learning Analysis (Evaluation 3—Machine Learning)

**Figure 1:**
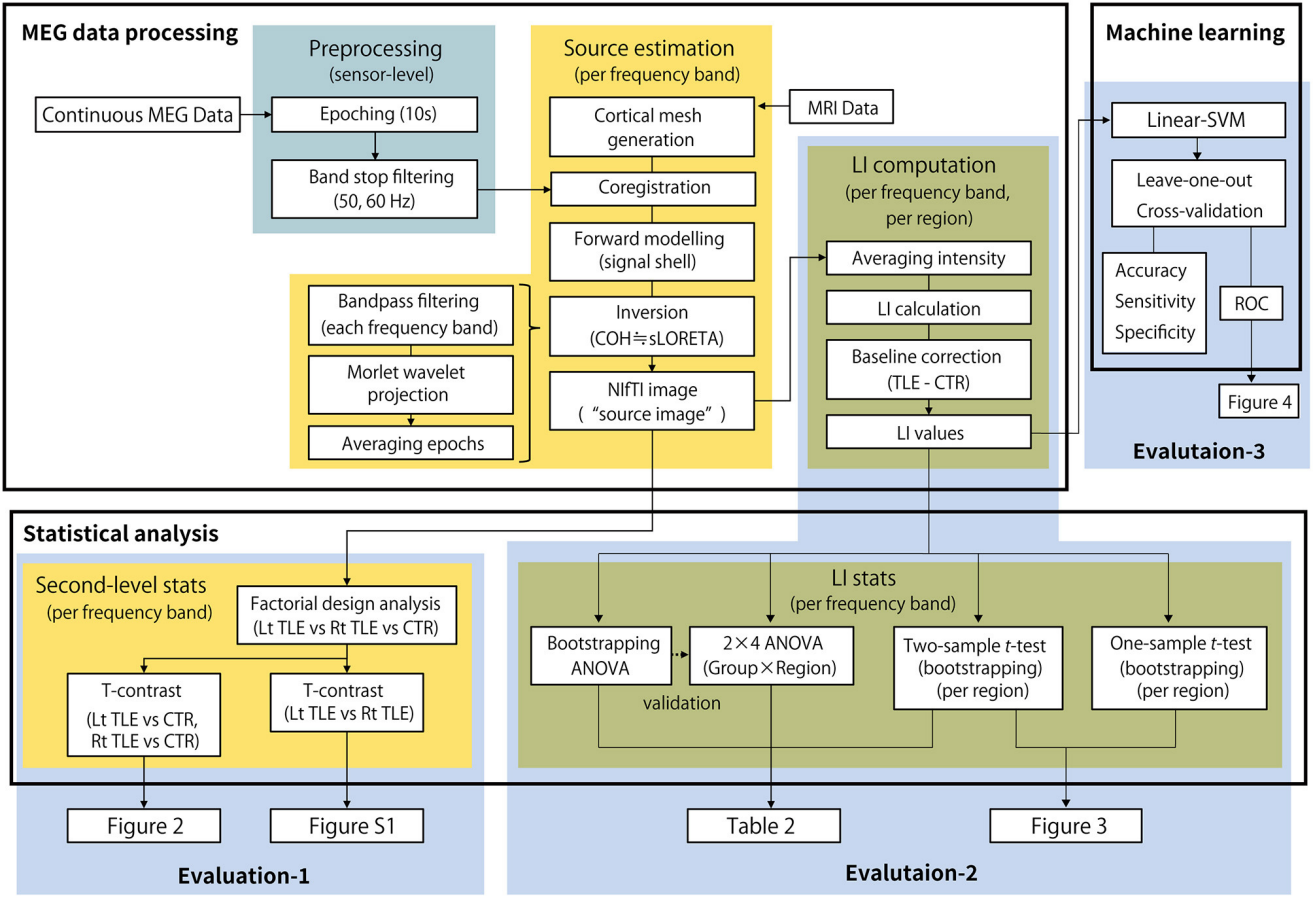
The summarized pipeline of magnetoencephalography data processing and statistical analysis. MEG, magnetoencephalography; LI, laterality index; Lt, left; Rt, right; TLE, temporal lobe epilepsy; CTR, healthy control; ANOVA, analysis of variance.

Finally, to ensure the predictability of LI for determining the side of the epileptic focus, a machine learning approach was applied. Following previous studies that applied machine learning techniques to localize epileptic focus using MEG data (31, 32), a support vector machine with linear kernel function (linear-SVM) was employed as a representative classifier. The linear-SVM classifier was trained to make a binary classification of the dataset between the groups in two scenarios: LtMTLE vs. CTR and RtMTLE vs. CTR. The regional LIs for each of the seven frequency bands (28 parameters) were standardized over the dataset and used as predictors. Since the number of samples in the MTLE groups were not large, and there was a large difference in the numbers between the groups (16 LtTLEs, 19 RtTLEs, and 102 CTRs), a leave-one-out cross-validation strategy was used to examine the predictability of classifiers in each scenario. In the validation, the training was performed using all samples except for one sample, which was reserved for the test data. The process was iterated for a number of samples [e.g., in the LtMTLE vs. CTR scenario, 16 (LtMTLE) + 102 (CTR) = 118 iterations], where different samples were reserved for the test data in each iteration. The result of cross-validation was summarized as a confusion matrix, where the predicted class for each sample was categorized into four types: true positive (TP), true negative (TN), false positive (FP), and false negative (FN). The matrix was then used to calculate the accuracy [(TP + TN)/(TP + FP + TN + FN)], sensitivity [TP/(TP + FN)], and specificity [TN/(FP +TN)]. To evaluate the characteristics of each classification scenario and model, a receiver operator curve (ROC) was drawn using the posterior probability (33) computed for each sample. The area under the ROC curve (AUC) was also estimated using the trapezoidal approximation. Statistical analyses and machine learning analyses were performed using the Statistics and Machine Learning Toolbox implemented in MATLAB (R2019b; Mathworks, Natick, MA, USA). The summarized pipeline for these analyses is shown in Figure 1.

## Results

### Oscillatory Power Differences Between MTLE Patients and Normal Healthy Controls

**Figure 2:**
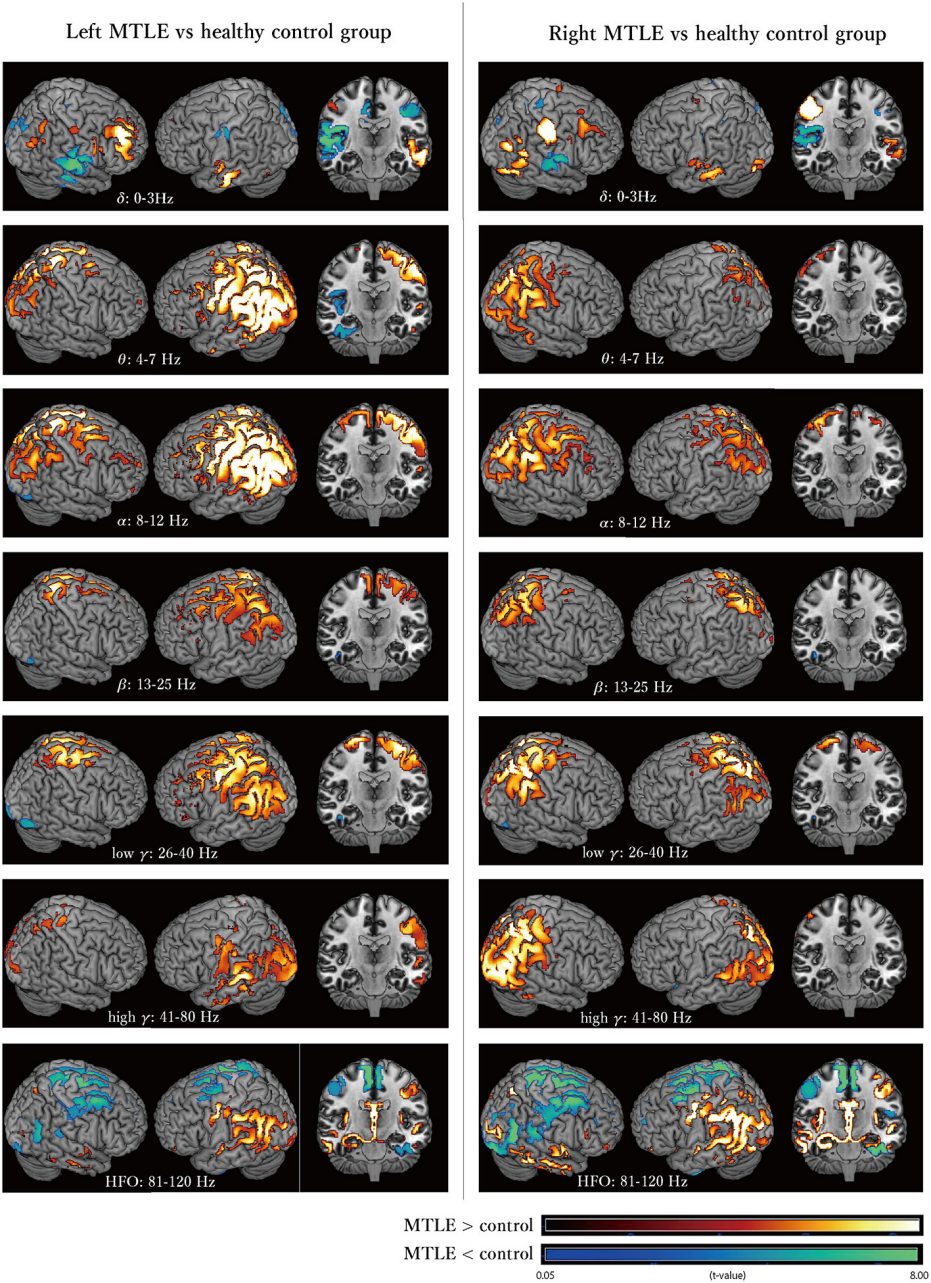
The cortical oscillatory powers in the LtMTLE group and the RtMTLE group in each frequency band (delta: 0–3 Hz, theta: 4–7 Hz, alpha: 8–12 Hz, beta: 13–25 Hz, low gamma: 26–40 Hz, high gamma: 41–80 Hz, and high frequency oscillation: 81–120 Hz) compared with CTR. The red color scale shows that the signal strength of the MTLE group is higher, and the green color scale shows that the signal strength of the MTLE group is lower. Significant oscillatory power differences between MTLE and CTR are seen throughout all frequency bands. In the theta, alpha, and high-gamma bands, there are marked increases of the oscillatory power at the parietal lobe, especially on the right side in RtMTLE. In the theta to HFO bands, there are marked increases at the parietal lobe, especially on the left side in LtMTLE. Frequency powers are lower at bilateral high frontal areas and interhemispheric areas in the HFO band, in both LtMTLE and RtMTLE. Lt, left; Rt, right; MTLE, mesial temporal lobe epilepsy; CTR, healthy control; HFO, high-frequency oscillation.

Figure 2 shows the cortical oscillatory powers in LtMTLE and RtMTLE compared with CTR. Areas with significantly higher or lower oscillatory powers were depicted on the normalized template brain model using MRIcron. The red color scale showed that the signal power in the MTLE group was higher, and the green color scale showed that the signal power in the MTLE group was lower. Throughout all frequency bands, there were areas with significant oscillatory power differences between the MTLE and CTR. In the theta to HFO bands, there were marked increases in the parietal lobe, especially on the left side, in LtMTLE. In the theta, alpha, and high-gamma bands, there were marked increases in oscillatory power at the parietal lobe, especially on the right side, in RtMTLE. Frequency powers were lower in the bilateral high frontal areas and interhemispheric areas in the HFO band, in both LtMTLE and RtMTLE. A comparison between LtMTLE and RtMTLE is shown in Supplementary Figure 1.

### Differences in LIs Between MTLE Patients and Normal Healthy Controls

**Figure 3:**
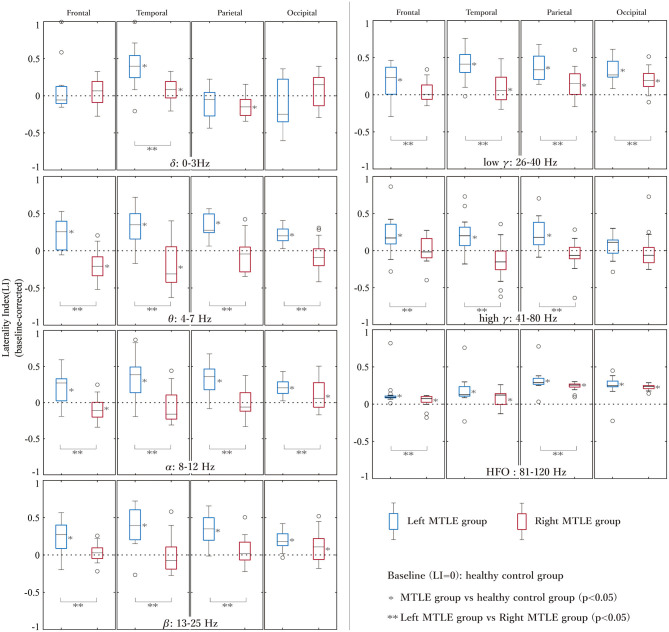
The LIs of the left and right MTLE groups at each brain region in each frequency band. LIs at each lobe (frontal, temporal, parietal, and occipital) and each frequency band are shown in the box-and-whiskers plots. LIs are significantly higher at 24/28 brain regions in LtMTLE, but lower at 4/28 regions and higher at 10/28 regions in RtMTLE. The LI at the temporal lobe in the theta band is significantly higher in LtMTLE and significantly lower in RtMTLE. Comparing LtMTLE and RtMTLE, there are significant LI differences at most brain regions in most frequency bands (21/28 regions). In all frequency bands, there are significant differences in LIs between LtMTLE and RtMTLE in the temporal and parietal lobes. LI, laterality index; Lt, left; Rt, right; MTLE, mesial temporal lobe epilepsy.

The baseline-corrected LIs of the LtMTLE and RtMTLE groups at each brain region, in each frequency band, are shown as box-and-whisker plots in Figure 3. The corresponding statistical values are presented in Table 2. Compared with CTR, LIs were significantly higher in 24/28 brain regions in LtMTLE, but lower in 4/28 regions and higher in 10/28 regions in RtMTLE. In particular, the LI at the temporal lobe in the theta band was significantly higher in LtMTLE and significantly lower in RtMTLE. Comparing LtMTLE and RtMTLE, there were significant LI differences in most brain regions in most frequency bands (21/28 regions). In all frequency bands, there were significant differences in the LI between the LtMTLE and RtMTLE, in the temporal and parietal lobes.

**Table 2:** Results of statistical analysis of LI.

	**ANOVA 2 (Group: LtMTLE and RtMTLE)** **×** **4 (Region: frontal, temporal, parietal, and occipital)**					**Two-sample** ***t*****-test** ** (LtTLE vs. RtTLE)**	
**Frequency**		***F***	***MS***	***p***	***p* (boot.)**	**Region**	***p* (boot.)**
δ	Main effect (Group)	3.74	0.322	0.055	0.052	Frontal	0.447
	^*^Main effect (Region)	7.43	0.639	<0.001	<0.001	^*^Temporal	<0.001
	^*^Interaction (Group^*^Region)	4.83	0.416	0.003	0.003	Parietal	0.046
						Occipital	0.094
θ	^*^Main effect (Group)	129.76	5.858	<0.001	<0.001	^*^Frontal	<0.001
	Main effect (Region)	1.38	0.062	0.251	0.247	^*^Temporal	<0.001
	Interaction (Group^*^Region)	2.64	0.119	0.052	0.051	^*^Parietal	<0.001
						^*^Occipital	<0.001
α	^*^Main effect (Group)	64.79	2.976	<0.001	<0.001	^*^Frontal	<0.001
	Main effect (Region)	1.88	0.086	0.136	0.137	^*^Temporal	<0.001
	^*^Interaction (Group^*^Region)	2.67	0.123	0.050	0.050	^*^Parietal	<0.001
						^*^Occipital	0.006
β	^*^Main effect (Group)	51.89	1.978	<0.001	<0.001	^*^Frontal	0.001
	Main effect (Region)	0.95	0.036	0.418	0.413	^*^Temporal	<0.001
	^*^Interaction (Group^*^Region)	3.57	0.136	0.016	0.017	^*^Parietal	<0.001
						Occipital	0.059
Low γ	^*^Main effect (Group)	44.76	1.451	<0.001	<0.001	^*^Frontal	0.014
	^*^Main effect (Region)	5.42	0.176	0.002	0.002	^*^Temporal	<0.001
	Interaction (Group^*^Region)	1.89	0.061	0.135	0.134	^*^Parietal	<0.001
						^*^Occipital	0.003
High γ	^*^Main effect (Group)	22.93	1.443	<0.001	<0.001	^*^Frontal	0.002
	Main effect (Region)	1.11	0.070	0.349	0.349	^*^Temporal	0.002
	Interaction (Group^*^Region)	1.16	0.073	0.328	0.333	^*^Parietal	0.004
						Occipital	0.110
HFO	^*^Main effect (Group)	10.17	0.165	0.002	0.002	^*^Frontal	0.001
	^*^Main effect (Region)	16.73	0.271	<0.001	<0.001	Temporal	0.040
	Interaction (Group^*^Region)	0.64	0.010	0.589	0.593	^*^Parietal	0.013
						Occipital	0.283

*ANOVA, analysis of variance; Lt, left; Rt, right; MTLE, mesial temporal lobe epilepsy; HFO, high-frequency oscillation; F, F statistics; MS, mean squares; p, p-value; bootstrap statistics*.

* *Statistically significant results after controlling for multiple comparisons using the FDR method*.

### Machine Learning-Based Classification

**Figure 4:**
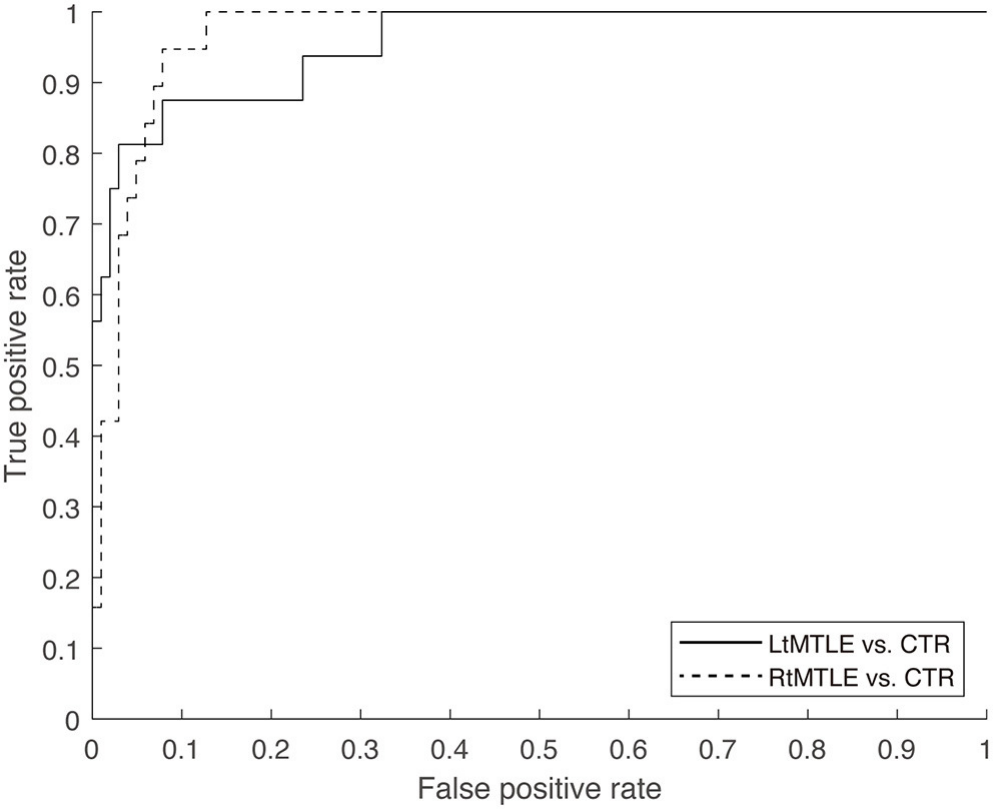
ROC of the linear-SVM classifier was shown for two scenarios (LtMTLE vs. CTR, RtMTLE vs. CTR). The AUCs were 0.96 for LtMTLE vs. CTR, and 0.97 for RtMTLE vs. CTR. ROC, receiver operator curve; SVM, support vector machine; Lt, left; MTLE, mesial temporal lobe epilepsy; CTR, healthy control; AUC, area under the curve.

The classification accuracy of the linear-SVM reached 94.1% (sensitivity, 75.0%; specificity, 97.1%; AUC, 0.96) for the LtMTLE vs. CTR classification scenario, and 91.7% (sensitivity, 68.4 %; specificity, 96.1%; AUC, 0.97) for RtMTLE vs. CTR. The models had high specificity and mild sensitivity, which was confirmed by visual inspection of ROCs (Figure 4). The estimated classes (i.e., side of the epileptic focus) during the leave-one-out cross-validation were listed in the Table 1. Among the 35 patients, the estimated lateralization of the 19 patients were corresponded between SVM classification and ECD method, those of 6 patients were found in SVM method alone, whereas those of 4 patients were found in ECD alone (the lateralization of remaining 6 patients were not specified neither by SVM nor ECD method).

## Discussion

### Summary of the Results

First, oscillatory power was compared between patients with unilateral MTLE and those with CTR in each frequency band. Compared to CTR, there were some brain areas with significantly higher and lower oscillatory powers. Oscillatory power was higher in the bilateral parietal lobes, especially on the ipsilateral side of the epileptic focus, in the theta to high-gamma ranges.

Second, oscillatory powers were compared between LtMTLE, RtMTLE, and CTR, using LIs in each brain region. Compared with CTR, LI was significantly higher in 24/28 lobes in LtMTLE, but lower in 4/28 lobes, and higher in 10/28 lobes in RtMTLE. Compared with RtMTLE and LtMTLE, the LIs were significantly higher in LtMTLE than in RtMTLE in most frequency bands and brain areas (21/28 regions). In the temporal lobe, LI in the theta band was significantly higher in the LtMTLE group and significantly lower in the RtMTLE group. The differences in LI between LtMTLE and RtMTLE were significant in all frequency bands, but prominent in the theta band.

Finally, the side of the epileptic focus was estimated using linear-SVM with regional LIs as predictors. The classification accuracy reached over 91%, where the model had high specificity over 96%, and mild sensitivity approximately 68–75%.

### Oscillatory Changes in Extratemporal Regions in MTLE

The present results showed that the differences in cortical oscillatory powers of MTLE patients were prominent in the parietal lobe, but not in the temporal lobe (Evaluation 1, Figure 2). There were significant LI differences in all frequency bands at the temporal lobe and parietal lobe, all but delta band at the frontal lobe, and theta, alpha, and low-gamma bands at the occipital lobe between the left and right MTLE (Evaluation 2, Figure 3). These results suggested that pathological oscillatory changes due to MTLE were not limited to the affected temporal lobe, but extended to other brain areas. Recently, Ricci et al. (34) reported that cortical activity is different between patients with temporal lobe epilepsy (TLE) and CTR, and that anti-epileptic medications reduce the gap between them, and regional connectivity from the epileptic focus and global connectivity values were reduced in patients achieving seizure freedom after medical treatment. These results also indicated that extratemporal regions, including the parietal lobe, were affected by the excitotoxic effects of epileptic activity in MTLE. The excitotoxic effects on extratemporal regions by TLE have also been reported in other studies (35–37). For example, in voxel-based morphology studies, widespread extratemporal cortical thinning was reported ipsilaterally (61.1%) and contralaterally (52.9%), and the locations of atrophy were mainly the thalamus, parietal lobe, and cingulate gyrus in TLE patients (35, 36). Cortical thinning was considered to be correlated with higher brain activity, such as cognitive function. The correlation between the hippocampus and temporoparietal lobe was discussed in a previous report that evaluated psychotic symptoms in patients with schizophrenia (37). In this report, the activities of the temporo-parieto-occipital junction were associated with hippocampal activity, and hippocampal over-activation stimulated other regions with excitotoxic activity. Moreover, brain atrophy in schizophrenia patients has been reported to be most frequently observed in the hippocampus and superior posterior temporal regions. Considering these reports, hippocampal activity is likely to affect the posterior temporal, parietal, and occipital lobes. The oscillatory power change at the parietal lobe observed in the present study can be caused by the excitotoxic activity of MTLE.

### Determination of Laterality Using LIs in Unilateral MTLE

From a practical clinical perspective, determining the side of the epileptic focus is important for MTLE. If there was a significant difference between unilateral MTLE patients and CTR subjects, it could be a valuable ‘lateralizing sign’. In a recent report regarding laterality diagnosis using MEG, the authors suggested that source imaging analysis using spatial filtering in TLE might be able to predict the laterality of the epileptic focus (21). The authors analyzed LIs in 14 TLE patients using beamformer analysis, and reported that the LI in the delta band was useful in determining the laterality in TLE, but they did not perform a comparison with a CTR group. As a clinical examination, it is common practice to compare patients' data with reference values acquired from a CTR group; thus, in the present study, the results of 35 patients with MTLE (left: 16 cases, right: 19 cases) and a CTR group composed of 102 healthy volunteers were compared. Furthermore, the present study confirmed the LI's predictability of the side of the epileptic focus using a machine learning approach. Compared to a previous study using graph-theory-based brain network metrics (nodal degree, betweenness centrality, and nodal efficiency) for predicting the lateralization of the epileptic focus of TLE (31), the present study achieved higher accuracies (over 91% against 88%) while using more datasets (137 against 30 samples). Present results also confirmed that the estimated classes of the linear-SVM reflected the side of the epileptic focus determined by the conventional ECD method (Table 1). Furthermore, those of 6 patients were specified by SVM method alone. This indicates that LIs provide promising information for determining the laterality of the epileptic foci. The present results suggest that the LIs, particularly in the theta band in the temporal lobe, could be a ‘lateralizing sign’ to determine the laterality of the epileptic focus; an LI from 0 to 1 indicates LtMTLE, and an LI from −1 to 0 indicates RtMTLE. The results obtained from the present study can offer new insights leading to an appropriate diagnosis of MTLE.

### Advantages of the Present Methods

Conventional MEG analysis for epilepsy is arbitrary and time-consuming, although it has produced excellent results (38–40). It does not work well for patients with few spikes. However, the method proposed here uses resting-state MEG data, which reflects the change in network connectivity, regardless of the presence of spikes (41, 42). The laterality of the epileptic focus was determined based on the regional frequency change due to epilepsy. It can be processed in an automated fashion using an ordinary personal computer in a short period (a maximum of a few hours). It does not rely on the skills of analysts, allowing them to engage in more important jobs that are cost effective. The present method does not require additional MEG recording, as it is applicable to MEG data recorded for conventional MEG analysis. It provides an additional perspective to help determine the side of the epileptic focus, and complements the conventional MEG analysis.

### Limitations

There are three potential limitations of the present study as follows: (i) We did not compare the reliabilities between the “lateralizing signs” provided by the present methods and other methods, such as conventional MEG analysis (i.e., ECD method) and other modalities (e.g., MRI and EEG). Medical diagnoses are essentially Bayesian processes; information is added up, where each of them improves the reliability of the final single diagnosis. All information can contribute toward improving it, even if the improvement is small (43, 44). The present methods were developed to complement the information provided by other methods/modalities to improve the final diagnosis, but not to replace them. From this perspective, there was no motivation to compare reliability across methods/modalities. (ii) Patients with various characteristics (duration of epilepsy, neuropsychological status, etc.) were included in the present study, which might have affected the results. Patients enrolled in the present study were typical unilateral MTLE patients diagnosed with seizure semiology and other modalities. However, in clinical situations, there are a number of patients in whom it is difficult to diagnose the laterality of the epileptic foci. In a future study, we plan to determine laterality using LIs in such clinically ambiguous situations and compare the results with those of other modalities. (iii) Only a minimal machine learning procedure was applied. Because LIs contain rich information (i.e., high dimensionality), they are suitable for the machine learning approach. Given the purpose of the present study, we only confirmed the predictability of the LIs using a linear-SVM with fixed parameters. In this minimal procedure, the training parameters (kernel functions, iterations, and input data) were not optimized, the classification scenarios were limited, and we did not examine whether the SVM would be the best classification model. Although the purpose of the present study was fulfilled using the minimal method, the validity of the machine learning approach must be analyzed further in our upcoming study.

## Conclusion

Using MEG frequency analysis, the characteristics of the oscillatory power distribution in the MTLE was demonstrated. Compared to CTR, LIs were shifted to the side of the epileptic focus at the temporal lobe in the theta band. The linear-SVM classification using LIs provided a high accuracy of over 91%. These results provide additional useful information for determining the laterality of epileptic foci.

## Data Availability Statement

The raw data supporting the conclusions of this article will be made available by the authors, without undue reservation.

## Ethics Statement

The studies involving human participants were reviewed and approved by the Ethics Committee of Hokuto Hospital (No. 1008) the Ethics Committee of Osaka City University Hospital (No. 4103). Written informed consent to participate in this study was provided by the participants' legal guardian/next of kin.

## Author Contributions

YT, TU, HH, and YS conceived and planned the experiments. HH and YS designed the model and the computational framework. YT, TU, YS, TK, KN, and NT contributed to sample preparation. YT, TU, HH, YS, NT, and TG contributed to the interpretation of the results. YT took the lead in writing the manuscript. All authors provided critical feedback and helped shape the research, analysis and manuscript.

## Conflict of Interest

The authors declare that the research was conducted in the absence of any commercial or financial relationships that could be construed as a potential conflict of interest.
