# Supplementary figures and images for: Specific Oscillatory Power Changes and Their Efficacy for Determining Laterality in Mesial Temporal Lobe Epilepsy: A Magnetoencephalographic Study
Source: Front Neurol. 2021 Feb 9;12:617291. doi: 10.3389/fneur.2021.617291 (PMC7900569; doi:10.3389/fneur.2021.617291)

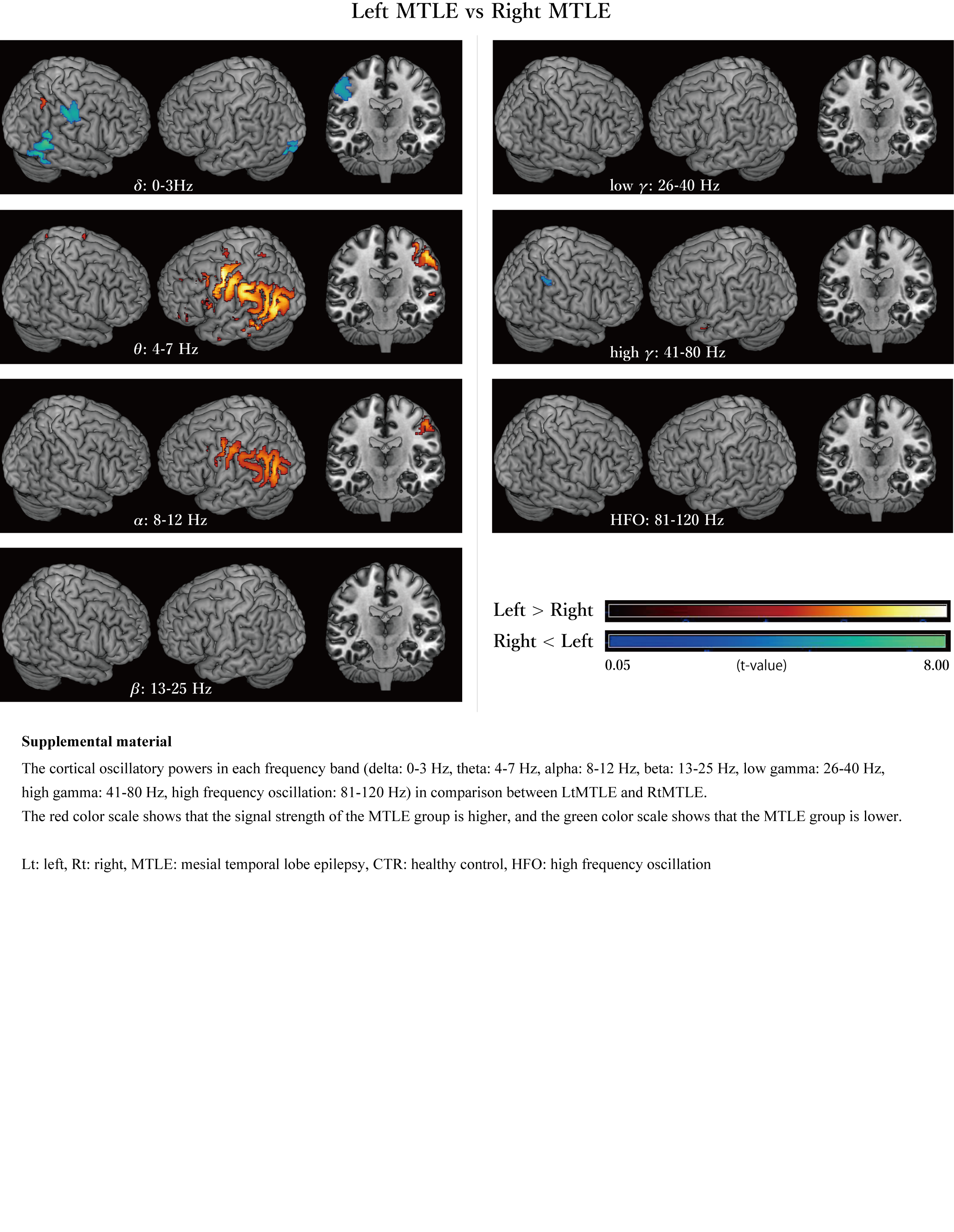

Supplement: Supplementary file 1 [file Image_1.TIF]
